# The Effect of Pupil Size on Cone Contrast Sensitivity

**DOI:** 10.3390/life15050801

**Published:** 2025-05-17

**Authors:** Ali Almustanyir, Meznah S. Almutairi, Amal Aldrwish, Nabeela Hasrod, Bader A. Alqhtani, Tahani Alqahtani, Muteb Alanazi, Mansour Alghamdi, Essam Almutleb, Balsam Alabdulkader, Faisal Fakhouri, Mosaad Alhassan

**Affiliations:** 1 Optometry Department, College of Applied Medical Sciences, King Saud University, Riyadh 11362, Saudi Arabia; mzalmutairi@ksu.edu.sa (M.S.A.); aaldarweesh@ksu.edu.sa (A.A.); 441100921@student.ksu.edu.sa (B.A.A.); talqahtani@ksu.edu.sa (T.A.); mkalanazi@ksu.edu.sa (M.A.); algmansour@ksu.edu.sa (M.A.); esalsarhani@ksu.edu.sa (E.A.); alabdulkader@ksu.edu.sa (B.A.); malhassan@ksu.edu.sa (M.A.); 2 Department of Optometry, Faculty of Health Sciences, University of Johannesburg, Johannesburg 2025, South Africa; nabeelah@uj.ac.za; 3 Department of Biomedical Technology, College of Applied Medical Sciences, King Saud University, Riyadh 11362, Saudi Arabia; ffakhouri@ksu.edu.sa

**Keywords:** pupil size, cone contrast sensitivity, wavelength cone systems, color vision

## Abstract

Background: Measuring contrast sensitivity for each of the three cone types separately allows for a more precise and clinically valuable assessment of color vision. This study examined how pupil size affects cone contrast sensitivity (CCS). Methods: This study included 50 participants of equal gender. The mean age was 20.88 (±1.8) years. Using the ColorDx, a Landolt C stimulus of various sizes in an adaptive screening mode, we sequentially determined contrast sensitivity for long-, medium-, and short-wavelength stimuli. Two consecutive measurements were performed on participants, one with their natural pupil size (range 4–5 mm diameter) and, subsequently, with six artificial eye pupils (1 mm to 6 mm). Results: Generally, the 1 mm pupil size caused the greatest reduction in contrast sensitivity for two of the three cones. There was no significant main effect of sex (F = 0.96, df = 1, *p* = 0.32) on the log cone contrast sensitivity of the L-cone. However, pupil size had a significant main effect (F = 116.1, df = 6, *p* < 0.001). Within each sex, the log CCS was significantly reduced as the pupil size decreased compared with the normal pupil size. Conclusions: New technologies that assess individual cone pathway functions could potentially assist in identifying early or progressive conditions that may impact color vision pathways from the retina to the brain. Standardized protocols—such as controlled retinal illumination—are critical to avoid misinterpretation.

## 1. Introduction

Color vision testing evaluates an individual’s capacity to perceive and differentiate various shades of color. These tests primarily diagnose color vision deficiencies, which affect approximately 8% of males [1]. Several types of color vision tests have been designed for specific purposes and varying levels of precision. For example, pseudoisochromatic plate tests are often employed to screen for inherited color vision disorders and exhibit high sensitivity [2]. However, these tests do have limitations. The determination of a specific type or severity of inherited color vision deficiencies is somewhat constrained, and tests rarely assess short-wavelength sensitivity. Furthermore, color plate tests are not commonly used in routine eye examinations, perhaps due to their limited ability to detect acquired color vision problems [3,4,5]. Notably, acquired color vision deficits may appear before the loss of high-contrast visual acuity or visual field defects [3,4,6].

Recent advancements in color perception testing technology have introduced methods for independently measuring color contrast thresholds for long (L), medium (M), and short (S) wavelength cone systems. This innovation enables the quantitative assessment of color perception without the need for specialized lighting conditions, which are required for color vision tests such as the Farnsworth D15 or 100-hue and pseudoisochromatic plate tests. It also removes the necessity for complex data interpretation generated by the gold-standard anomaloscope used in color-matching tests. Although pseudoisochromatic color plates, arrangement tests, matching tests, and contrast sensitivity tests evaluate different aspects of color perception, comparative studies involving individuals with normal vision and those with known inherited color vision deficiencies have demonstrated that these tests show comparable sensitivity and specificity in distinguishing between normal and anomalous trichromacy or dichromacy [6].

The cone contrast sensitivity (CCS) test, developed in the late 1990s, has been validated on military personnel [7]. In a 1996 study focusing on quantifying color perception using CCS, researchers employed a uniform stimulus size (6/95) for all three chromatic stimuli based on British Standard Institution letters [6]. This large stimulus size was specifically chosen to minimize the effect of defocus when viewing chromatic stimuli against an achromatic background [7]. It revealed that, for normal trichromatic vision, the CCS for the short-wavelength (S) stimulus was nearly one log unit lower than for the medium-wavelength cone (M) and long-wavelength cone (L) stimuli, respectively. This significant difference in sensitivity among the different cone types offers valuable insights into the varying sensitivities of the human visual system to different wavelengths [7,8].

In the 2011 test iteration, the S-wavelength stimulus was enlarged to 6/132 to align with the peak spatial frequency for the contrast detection of the S-wavelength neural mechanism [9]. This adjustment resulted in a higher CCS in individuals with normal trichromatic vision. Notably, using an S-wavelength stimulus larger than L- and M-wavelength stimuli is consistent with other studies investigating visual acuity and contrast sensitivity in cone-specific pathways utilizing letter charts and visually evoked potentials [10,11]. The enlarged S-wavelength stimulus also engaged a significant number of S-cones while remaining within the 3° macular region containing the macular pigment [12]. Additionally, in this version, each stimulus was displayed as a single letter on a monitor for 1.0–1.6 s.

As reported in 1996 and 2004, in comparing the CCS to other clinical color vision tests, including the gold-standard anomaloscope, the CCS test demonstrated comparable effectiveness in distinguishing between normal trichromacy and red–green anomalous trichromacy or dichromacy [7,8]. Moreover, the CCS test successfully identified defective color vision in patients with high-contrast acuity visual pathologies, sometimes detecting issues missed by other tests [9]. Konan Medical, Inc., in collaboration with the U.S. Air Force School of Aerospace Medicine, enhanced the original CCS clinical instrument by [13]:

- Expanding the evaluable contrast range;
- Substituting letter stimuli with Landolt C stimuli;
- Implementing a four-direction keypad for responses;
- Introducing an “adaptive screening mode” for efficiency.

Cisarik and Kampwerth evaluated the effects of pupil size on CCS using ColorDx technology (Konan Medical, Inc., Irvine, CA, USA) in young healthy adults [14]. They found that CCS decreased when retinal illumination was reduced in healthy individuals with normal color vision. This reduction was achieved by having the participants view through an artificial pupil smaller than their natural pupil size. Such effects are expected, as previous research has shown, that the thresholds for achromatic (black and white) contrast increase under conditions of reduced retinal illumination [15,16]. Notably, while a 2.5 mm pupil has been previously identified as optimal for enhancing contrast sensitivity and visual acuity, this study found that using an artificial pupil of this size negatively affected the CCS of participants. Their performance was better when using their larger natural pupils compared to the artificially created 2.5 mm pupils. Cisarik and Kampwerth suggested that the relationship between pupil size and color perception is complex and that what may be optimal for some aspects of vision (such as achromatic contrast sensitivity and visual acuity), such as cone contrast sensitivity, may not be ideal for others. Nevertheless, this study extended previous works to evaluate the effect of different retinal illumination sizes on CCS assessed using ColorDx technology (Konan Medical, Inc.) in normal adults.

Previous studies have also suggested that, in conditions such as early glaucoma, diabetic retinopathy, and optic neuropathies, contrast sensitivity deficits may precede measurable changes in high-contrast visual acuity or visual fields [17,18].

By having a better understanding of the effect of pupil size on cone contrast sensitivity, this study could potentially help clinicians with a better interpretation of contrast sensitivity tests specifically in non-standard lighting conditions, or in the presence of miosis or mydriasis, which could help reduce false positives/negative findings in early retinal or optic nerve dysfunction diagnosis.

Similarly, when assessing for early retinal or optic nerve dysfunction, as senile miosis may influence cone contrast responses, the findings could guide clinicians’ expectations in older adults [19].

This study may also lead to developing best practices for clinicians for pupil control and ambient lighting during cone contrast sensitivity testing.

Knowing the impact of pupil size on contrast sensitivity could also help manage patients’ expectations of visual quality under different lighting conditions when wearing multifocal contact lenses or post refractive surgery [20,21]. This study aims to evaluate the effect of different retinal illumination sizes on the CCS assessed with the ColorDx technology (Konan Medical, Inc.) in normal adults.

## 2. Materials and Methods

This study was approved by the Institutional Review Board (IRB) of the College of Medicine at King Saud University, Saudi Arabia. This study followed the Declaration of Helsinki’s tenets and was approved by King Saud University through the Institution Review Board Office of Research Ethics (E-23-8128). After obtaining written informed consent as per the IRB-approved protocol, a group of 25 male and 25 female healthy young adults between the ages of 21 and 31 took part in the study. All participants had undergone different color vision tests. All subjects were found to have normal trichromatic vision. All participants had healthy visual systems. The use of tinted contact lenses or glasses was not permitted, and any history of eye disease was ruled out through a brief questionnaire. To further minimize the risk of including individuals with bilateral disorders linked to acquired color vision defects, only those with binocular distance visual acuity of 6/9 or better were selected. Presbyopic participants wore their usual near correction during testing.

### 2.1. Instrumentation

Pseudoisochromatic plate tests were used to test the normality of color vision. The pseudoisochromatic plate tests used were the 38-plate edition of the Ishihara test (Kanhara & Co., Ltd., 1996, Tokyo, Japan) and the Hardy, Rand, and Rittler tests (4th edition, Richmond Products, Albuquerque, NM, USA). The normality of individual color vision was classified according to Rayleigh color matching using an HMC Oculus anomaloscope (Oculus Optikgeräte GmbH, Wetzlar, Germany). Tinted contact lenses or spectacles were not used in this study. The sequences of all tests were randomized using a random block design. The failure criterion for the Ishihara screening plates was ≥3 errors on plates 1–17, >2 errors on the red–green screening plate, and >0 errors on the blue–yellow plates of HRR. The illuminance for the tests was 800 lux (±5%) in the horizontal plane. The ColorDx test was performed to measure the CCS of all cones (L, M, and S). Tropicamide eye drops (1%) were used to control pupil size. The drop was instilled 30 min before the examination started.

The test was repeated for different pupil sizes (1–6 mm) using artificial pupils.

**Figure 1:**
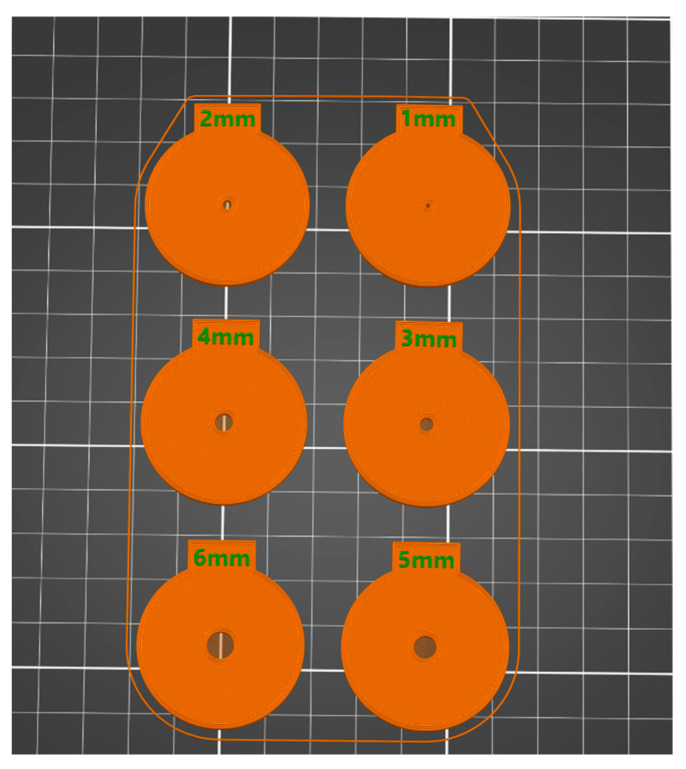
Illustration of the artificial pupil sizes used in this study. There were 6 sizes (1 to 6 mm) in 1 mm steps. The diameter of each of the artificial pupils was 37 mm. Each artificial pupil was marked with the pupil size at the top in mm.

Six circular artificial pupils were 3D-printed with the same outer diameter of 37 mm and a thickness of 2 mm. Each artificial pupil had a hole in the center of the circle with a diameter ranging from 1 to 6 mm, as shown in Figure 1. Each artificial pupil was installed on the trial frame on the right eye, and the left eye was covered by the frame occluder. 3D printing was performed using a Prusa MK3S+ (Prusa Research Inc., Prague, Czech Republic) fused deposition modeling (FDM) 3D printer. The 3D-printed objects were printed using an opaque dark blue eSun PLA+ filament (Shenzhen Esun Industrial Co., Ltd., Shenzhen, China), a poly lactic acid-based (PLA) 3D printing material. The 3D printing parameters were as follows: layer height 0.1 mm, infill percentage 100%, printing nozzle temperature 210 °C, printing bed temperature 60 °C with a smooth finish, and a printing time of 2 h and 52 min.

The ColorDx test was used to evaluate CCS. This system comprises proprietary software (version 1.0.079), a Dell all-in-one computer with a 21.5-inch FHD LED-backlit IPS monitor, a 4-arrow response keypad, and a colorimeter for periodic screen calibration [22,23]. The software presents Landolt-C stimuli using the Bayesian [24] adaptive technique to determine the thresholds and standard errors. This technique adaptively targets nuisance parameters only when optimizing the estimation of the primary parameters of interest [24]. The monitor, with a maximum resolution of 1920 × 1080 pixels and a 60 Hz refresh rate, features an anti-glare color-calibrated screen with wide viewing angles. Before data collection, the monitor was calibrated using an OEM i1Pro USB colorimeter (Konan Medical). During testing, the luminance of the monitor was set to 65 candela/square meter, as measured by a Konica Minolta LS-150 luminance meter.

### 2.2. Procedure

**Figure 2:**
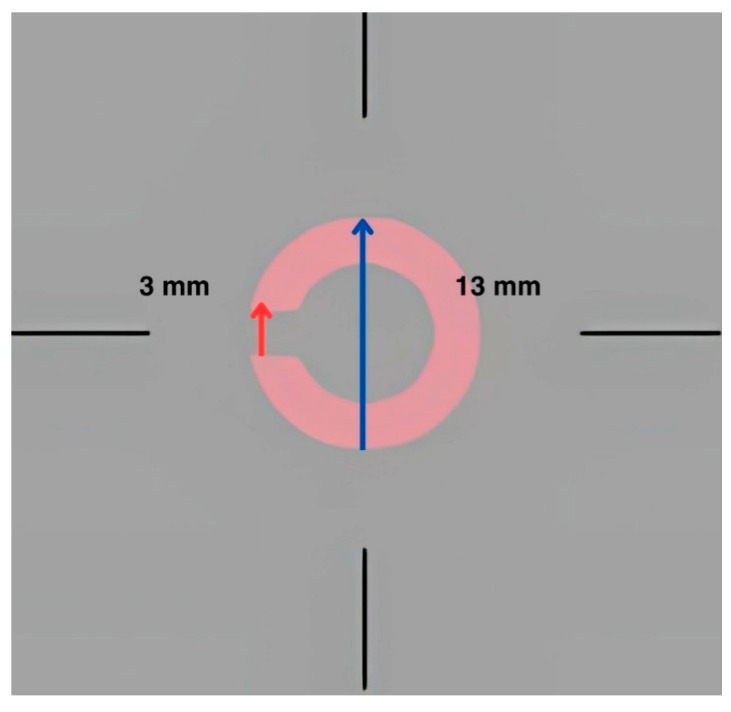
A screenshot of the Landolt C that was used in the CCS test for the L cone. Red arrow indicate the size of the opening gap of the Landolt C, and the blue arrow indicates the size of the Landolt C.

The target was Landolt C, and participants needed to determine the direction of the letter ‘C’. The viewing distance was 40 cm, which subtended 1.8° at the retina. The letter ‘C’ appears in the center of the screen, oriented in a specific direction. The options faced upward, downward, right, or left. The participants pressed the arrow key corresponding to the direction they perceived. The stimulus (letter) remained visible for 5 s before it disappeared. Whenever the participant answered correctly, the contrast decreased, and vice versa. Next, the tropicamide eye drops were administered to the right eye three times. Five minutes were spent waiting between the first and second drops, and the third drop after 15 min. The participants waited until the eye expanded completely (at least 45 min). The next step was to randomly place the artificial eye pupil on a trial frame (if the participant had a correction, we placed the prescription on the trial). There was a 1–2 min rest between each measurement. This was completed to ensure that the cones (L, M, and S) were fatigued. Figure 2 shows an example of a Landolt C presented. The Logarithm (Log) CCS was one of the outcomes from the test. Generally, the Log CCS is a logarithmic measure of the eye’s ability to detect differences in contrast between an object and its background based on color, quantifying how well a person can see objects that do not stand out clearly from their surroundings.

### 2.3. Statistical Analysis

Repeated measures analysis of variance (ANOVA) was employed to investigate the impact of sex and pupil size on log cone contrast sensitivity (log CCS). The mean, standard deviation of the mean, and the 95% confidence interval were calculated. The statistical significance level was set at 5% (*p* < 0.05). All statistical analyses were conducted using IBM SPSS Statistics version 29 (IBM, Armonk, NY, USA).

## 3. Results

The participants’ mean age was 20.88 (±1.8) years. There were 50% male and 50% female participants. Natural pupil sizes ranged from 4 to 5 mm. All participants tested with their natural pupils demonstrated normal trichromacy, as indicated by their CCS measurements, pseudoisochromatic plate tests, and anomaloscope.

A two-way mixed-effects ANOVA was conducted to investigate the impact of sex and pupil size on log cone contrast sensitivity (log CCS). There was no significant main effect of sex (F = 0.96, df = 1, *p* = 0.32) on the log CS of the L-cone. However, pupil size had a significant main effect (F = 116.1, df = 6, *p* < 0.001). Within each sex, the log CCS was significantly reduced as the pupil size decreased compared with the normal pupil size (Table 1). The pairwise comparison test showed a significant difference between the genders with a 2 mm pupil size (mean difference = 0.086, *p* = 0.043).

Similarly, there was no significant main effect of sex on the log CCS of M-cones (F = 1.35, df = 1, *p* = 0.25; Table 2) or S-cones (F = 0.198, df = 1, 0.66; Table 3). Pupil size had a significant effect on the M-cone (F = 131.2.1, df = 6, *p* < 0.001) and S-cones (F = 125.3, df = 6, *p* < 0.001). The pairwise comparison test showed a significant difference between the genders with a 1 mm pupil size in the M-cone (mean difference = 0.106, *p* = 0.041). No significant difference was found between males and females using a multiple comparison test for the S-cone.

**Figure 3:**
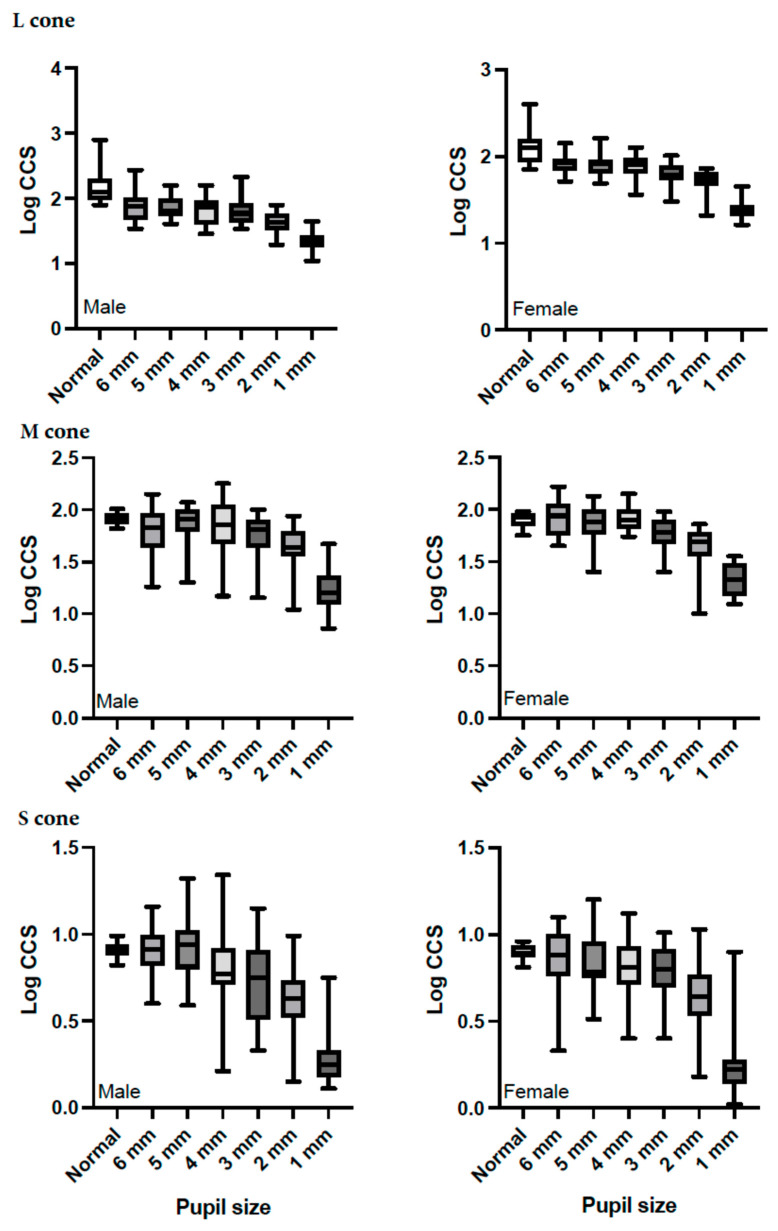
Boxplots showing the log cone contrast sensitivity (CCS) data (female: right figures and male: left figures) for each chromaticity stimulus (top: L-cone; middle: M-cone; last: S-cone) displayed at six pupil sizes. Results obtained with the natural pupil are presented as (Normal).

Figure 3 provides the descriptive statistics for the log CCS measurements for each cone stimulus for the six different pupil sizes. Boxplots present the CCS data for the six pupil sizes for the L-cone (Figure 3 top), M-cone (Figure 3 middle), and S-cone (Figure 3 bottom) stimuli. A reduction in log CCS occurred with the smallest pupil size (1 mm) for all three types of cones. The CCS increased as pupil size increased across the three types of cones.

## 4. Discussion

In healthy participants with normal color vision, reducing retinal illumination using an artificial pupil smaller than their natural pupil size led to a significant decline in cone contrast sensitivity (CCS). This aligns with the known effects of lower illumination on the achromatic contrast thresholds, which also increase under dimmer conditions [15,16]. Specifically, this study showed that, generally, the 1 mm pupil size caused the greatest reduction in contrast sensitivity for two of the three cones (L-cone: [0.703: 0.84]; M-cone [0.566: 0.691]).

Data analysis employing two-way mixed-effect ANOVA and boxplots illustrated that, within each sex, the log CCS was significantly reduced as the pupil size was reduced compared to the normal pupil size for all three chromaticities. Pupil size had a significant main effect (F = 116.1, df = 6, *p* < 0.001) on the log CCS of L-cone, M-cone (F = 131.2.1, df = 6, *p* < 0.001), and S-cones (F = 125.3, df = 6, *p* < 0.001).

Generally, although there was no significant main effect of gender on the log CS of the L-cone (F = 0.96, df = 1, *p* = 0.32), M-cone (F = 1.35, df = 1, *p* = 0.25, Table 2), and S-cone (F = 0.198, df = 1, 0.66, Table 3), the pairwise comparison test did show a significant difference between the genders with 2 mm pupil size (mean difference = 0.086, *p* = 0.043) for the log CS of L-cone, and with 1 mm pupil size in the M-cone (mean difference = 0.106, *p* = 0.041).

Regarding the reduction in log CCS across all three chromaticities, it was found that the 1 mm pupil size produced the greatest mean differences between the normal for the L-cone (male: 0.84; female: 0.703) and M-cone (male: 0.691; female: 0.566). However, for the S-cone, the 2 mm pupil size produced the greatest mean difference from the normal (male: 0.623; female: 0.609).

The practical implications of the 1 mm pupil size and a measurable decrease in log CCS have several practical implications for clinicians. When measuring elderly patients or those with pharmacologically constricted pupils, where smaller pupil sizes may artificially lower scores, this finding emphasizes the importance of considering pupil size when interpreting contrast sensitivity test results. Also, clinicians should consider these implications when administering miotic drugs (e.g., pilocarpine) or testing contrast sensitivity under non-standard lighting conditions, as these can reduce pupil diameter and potentially mask true visual function. Finally, understanding this relationship may help manage the expectations of patients with underlying neural or retinal disorders where contrast sensitivity is already compromised. This reiterates the value of assessing both structural and functional vision, including contrast sensitivity, as part of a holistic visual evaluation [17,18,25].

Although the normal or natural pupil size ranged from 4 mm to 5 mm for all participants, it was interesting to note that, with a few exceptions (M-cone for females and S-cones for males), generally, the mean log CCS produced by the normal pupil for both sexes was still greater than the log CS for the 4 mm and 5 mm artificial pupil sizes for all chromaticities. It is possible that, for artificial pupils created by apertures placed in the spectacle plane, field-of-view restrictions may result from vignetting with the eye pupil. Thus, smaller artificial pupil sizes can act as pinholes, causing the target resolution to become impaired, which can adversely affect CCS [26].

Nevertheless, when comparing results from this study to that by Cisarik and Kampwerth with a 2.5 pupil size for all chromaticities (L-cone: large stimulus: 0.309, small stimulus: 0.260; M-cone: large stimulus: 0.297, small stimulus: 0.273; S-cone: large stimulus: 0.163, small stimulus: 0.197), it was found that, for the L-cone and S-cone, results were similar to the mean difference in log CCS between the normal for the 3 mm pupil size for both sexes (L-cone: male: 0.394, female: 0.294; S-cone: male: 0.292, female: 0.211). Regarding M-cones, the results were similar for the 2 mm pupil size for both sexes (male: 0.280, female: 0.261) [14].

It can be seen that the S-cone, generally, had smaller mean Log contrast sensitivity for all pupil sizes as compared to M- and L-cone sensitivities. One reason for this could be longitudinal chromatic aberration. With larger pupils, peripheral rays are affected, and there is an increase in optical blurring and light scattering. This worsens the chromatic aberrations, thus reducing contrast sensitivity [27].

The smallest artificial pupil size (1 mm) reduced retinal illumination by approximately 75%. This shifted the testing conditions from photopic to mesopic retinal light levels. Nevertheless, the 75% decrease from the original monitor luminance of 65 cd/m² to 16 cd/m² remained within the photopic range (defined as >3.0 cd/m²) [28]. Under these conditions, the rod cells likely play a negligible role in detecting S-wavelength stimuli. This minimal rod involvement holds across all tested stimulus sizes because photopic vision (dominated by cone cells) typically suppresses rod activity in bright environments.

Standardized and documented testing conditions are essential to ensure that visual function tests can effectively track disease progression [29]. Although a patient’s natural pupil size under consistent lighting remains relatively stable over time, clinicians must account for variations in retinal illumination caused by pupil dilation. For instance, when monitoring diabetic retinopathy progression via CCS, comparing the results from a dilated pupil visit versus those from an undilated visit could lead to a misinterpretation of retinal health. Differences in illumination may artificially alter the CCS scores, potentially masking or exaggerating disease-related changes. Thus, clinicians should maintain consistent protocols (e.g., controlling the dilation status) to avoid false positive diagnostic errors.

## 5. Strengths, Limitations, and Recommendations

The findings of this study were limited by the homogeneous age range of the participants. Age-related retinal dysfunction, which can affect color perception, and cataract development, which reduces retinal illuminance, are more common in older populations. It is essential to extend this study to include older adults with healthy retinas and visual pathways [30]. Future research should encompass a broader age range and include populations with retinal or visual pathway disorders. This approach would enhance our understanding of how retinal illuminance and stimulus size influence clinical contrast sensitivity (CCS) measurements. Such studies would improve the reliability of CCS as a diagnostic tool and help distinguish between changes related to aging, disease, or testing artifacts. Despite these limitations, this study provided valuable insights into how pupil size interacts with CCS. This information is particularly important in aging eyes that experience natural lens changes and illumination deficits, as it is critical for adopting CCS as a screening tool for retinal or neural visual pathway disorders in older individuals. Without this understanding, age-related physiological changes could obscure or mimic pathological trends, compromising the diagnostic accuracy of the test in this demographic group.

## 6. Conclusions

This study evaluated the impact of different retinal illumination sizes on cone contrast sensitivity (CCS) using ColorDx technology. The results contributed to the development of optimal guidelines for clinicians regarding pupil management and ambient lighting conditions during CCS testing. Additionally, emerging technologies that assess individual cone pathway functions show promise for detecting early or progressive pathologies that affect color vision pathways from the retina to the brain. To reduce the risk of data misinterpretation, it is recommended to use standardized testing conditions, including consistent retinal illumination.

## Figures and Tables

**Table 1:** The mean Log contrast sensitivity (log CCS) of the L-cone.

Gender	Pupil Size (mm)	Mean ± SD	95% Confidence Interval	Mean Difference from Normal	*p*-Value
Males	Normal	2.183 ± 0.044	2.095–2.270	—	—
	1 mm	1.341 ± 0.025	1.292–1.390	0.842	<0.001
	2 mm	1.641 ± 0.028	1.585–1.697	0.542	<0.001
	3 mm	1.789 ± 0.032	1.724–1.853	0.394	<0.001
	4 mm	1.812 ± 0.033	1.746–1.878	0.371	<0.001
	5 mm	1.861 ± 0.028	1.805–1.917	0.322	<0.001
	6 mm	1.872 ± 0.035	1.801–1.943	0.311	<0.001
Females	Normal	2.092 ± 0.051	1.989–2.195	—	—
	1 mm	1.389 ± 0.029	1.331–1.447	0.703	<0.001
	2 mm	1.726 ± 0.033 *	1.660–1.792	0.366	<0.001
	3 mm	1.798 ± 0.037	1.722–1.873	0.294	<0.001
	4 mm	1.880 ± 0.039	1.803–1.958	0.211	0.026
	5 mm	1.905 ± 0.033	1.839–1.971	0.187	0.050
	6 mm	1.910 ± 0.042	1.827–1.994	0.181	0.089

The 95% confidence interval, mean difference from normal, and *p*-value are presented. * Significant mean difference in log CCS in the females compared to males (*p* = 0.043).

**Table 2:** The mean Log contrast sensitivity (log CCS) of the M-cone.

Gender	Pupil Size (mm)	Mean ± SD	95% Confidence Interval	Mean Difference from Normal	*p*-Value
Males	Normal	1.917 ± 0.012	1.893–1.942	—	—
	1 mm	1.226 ± 0.033	1.160–1.292	0.691	<0.001
	2 mm	1.637 ± 0.037	1.563–1.710	0.280	<0.001
	3 mm	1.768 ± 0.031	1.705–1.831	0.149	<0.001
	4 mm	1.832 ± 0.037	1.756–1.907	0.086	0.037
	5 mm	1.853 ± 0.037	1.779–1.928	0.064	0.120
	6 mm	1.819 ± 0.036	1.746–1.891	0.099	0.013
Females	Normal	1.899 ± 0.014	1.870–1.928	—	—
	1 mm	1.332 ± 0.039 *	1.255–1.410	0.566	<0.001
	2 mm	1.638 ± 0.043	1.551–1.724	0.261	<0.001
	3 mm	1.779 ± 0.037	1.704–1.853	0.120	0.007
	4 mm	1.919 ± 0.044	1.831–2.007	−0.020	0.664
	5 mm	1.847 ± 0.043	1.759–1.934	0.052	0.279
	6 mm	1.930 ± 0.043	1.845–2.016	−0.032	0.481

The 95% confidence interval, mean difference from normal, and *p*-value are presented. * Significant mean difference in log CCS in the females compared to males (*p* = 0.041).

**Table 3:** The mean Log contrast sensitivity (log CCS) of the S-cone.

Gender	Pupil Size (mm)	Mean ± SD	95% Confidence Interval	Mean Difference from Normal	*p*-Value
Males	Normal	0.904 ± 0.029	0.845–0.963	—	—
	1 mm	0.907 ± 0.008	0.892–0.922	−0.003	1.0
	2 mm	0.280 ± 0.03	0.220–0.341	0.623	<0.001
	3 mm	0.612 ± 0.038	0.535–0.689	0.292	<0.001
	4 mm	0.713 ± 0.039	0.635–0.792	0.190	<0.001
	5 mm	0.810 ± 0.036	0.737–0.883	0.094	0.29
	6 mm	0.910 ± 0.031	0.853–0.979	−0.012	1.0
Females	Normal	0.851 ± 0.034	0.782–0.921	—	—
	1 mm	0.899 ± 0.009	0.881–0.916	−0.047	1.0
	2 mm	0.242 ± 0.035	0.171–0.314	0.609	<0.001
	3 mm	0.640 ± 0.045	0.549–0.731	0.211	<0.001
	4 mm	0.771 ± 0.046	0.679–0.864	0.080	1.0
	5 mm	0.798 ± 0.043	0.712–0.883	0.054	1.0
	6 mm	0.841 ± 0.037	0.768–0.915	0.010	1.0

The 95% confidence interval, mean difference from normal, and *p*-value are presented.

## Data Availability

Data are contained within the article.
